# Machine Learning in Predicting the Risk of Esophagogastric Variceal Bleeding Among Patients With Liver Cirrhosis: Systematic Review and Meta-Analysis

**DOI:** 10.2196/78203

**Published:** 2026-04-08

**Authors:** Yuan Lian, Xinping Qiu, Geli Wang, Nannan Liu, Jing Zhao, Shanshan Song, Shiqi Wang, Mingjun Sun

**Affiliations:** 1 Department of Gastroenterology, Fuxin Mining General Hospital of Liaoning Health Industry Group, No.43,Health Care Street,Haizhou District, Fuxin, China, 86 18341893085; 2 Department of Gastroenterology, The First Hospital of China Medical University, Shenyang, China

**Keywords:** liver cirrhosis, hepatic, prediction model, esophageal variceal bleeding, esophagogastric variceal bleeding, radiomics, machine learning

## Abstract

**Background:**

Liver cirrhosis (LC) can lead to several complications. Esophageal variceal bleeding (EVB) and esophagogastric variceal bleeding (EGVB) are particularly severe, leading to a high risk of mortality. Early identification of esophageal varices and esophagogastric varices is essential. Several studies have constructed prediction models for EVB and EGVB in patients with LC. However, robust systematic evidence to prove their performance is lacking.

**Objective:**

We included original studies that developed prediction models for esophageal or gastric variceal bleeding in patients with LC under different modeling variables. This study aimed to review the predictive performance of various models for EVB or EGVB in patients with LC, providing insights into the development or updating of simplified scoring tools in the future.

**Methods:**

PubMed, Web of Science, Embase, and the Cochrane Library were searched up to August 21, 2024, to collect original full-text studies on machine learning (ML) in the prediction of EVB and EGVB in patients with LC. The models were evaluated using the Prediction Model Risk of Bias Assessment Tool (PROBAST). Subgroup analyses were carried out based on the modeling variables.

**Results:**

In total, 21 studies were included, with 7011 patients with LC, among whom 1412 (20.14%) developed EVB and 733 (10.45%) developed EGVB. The meta-analysis results suggested that the pooled c-index, sensitivity, and specificity of the prediction model for predicting EVB in the validation set were 0.85 (95% CI 0.77‐0.92), 0.93 (95% CI 0.87‐0.96), and 0.66 (95% CI 0.46‐0.82), respectively. The pooled c-index, sensitivity, and specificity of the prediction model for predicting EGVB in the validation set were 0.89 (95% CI 0.85‐0.94), 0.77 (95% CI 0.66‐0.85), and 0.81 (95% CI 0.67‐0.90), respectively. The subgroup analysis based on modeling variables revealed that, for predicting EVB, the c-index in the validation set was 0.84 (95% CI 0.80‐0.88) for models based on clinical features, 0.82 (95% CI 0.69‐0.96) for radiomics-based models, 0.78 (95% CI 0.67‐0.89) for models based on radiomics and clinical features, and 0.97 (95% CI 0.95‐1.00) for models based on endoscopic features. Subgroup analyses based on modeling variables revealed that, for predicting EGVB, the c-index in the validation set was 0.91 (95% CI 0.86‐0.96) for models based on clinical features and 0.85 (95% CI 0.75‐0.96) for models based on radiomics and clinical features.

**Conclusions:**

ML methods are feasible for predicting EVB and EGVB in patients with LC. Nevertheless, the number of included original studies is limited. In the future, more studies with larger sample sizes are needed to promote the application of ML in the early assessment of EVB and EGVB in patients with LC in clinical practice.

## Introduction

Liver cirrhosis (LC) often occurs after long-term inflammation, resulting in fibrotic tissue and regenerative nodules and, subsequently, portal hypertension [1]. Approximately 1 million deaths worldwide are attributable to LC every year [2]. LC is the 11th leading cause of death worldwide and the third leading cause of death among individuals aged 45 to 64 years. Together with liver cancer, it represents 3.5% of global deaths [2]. LC contributed to 2.4% of deaths worldwide in 2019 [3]. LC is a notable cause of both morbidity and mortality in patients with chronic liver diseases, leading to hepatocellular carcinoma (HCC) and liver decompensation. LC-induced portal hypertension is associated with complications such as ascites, hepatic encephalopathy, acute kidney injury, hepatorenal syndrome, and portal hypertension–related bleeding, such as esophagogastric variceal bleeding (EGVB) [1]. Thus, LC becomes a major health care burden worldwide.

The incidence of EGVB is high among the primary complications of LC. It is the second most prevalent complication in patients with LC, after ascites. EGVB, a frequent cause of bleeding, is regarded as a medical emergency. This complication, even when well managed, is associated with a mortality rate of approximately 20% within 6 weeks. If an infection occurs, the risk of mortality increases further [1]. EGVB is a critical condition and often recurs. In severe cases, it may be life threatening [4]. Currently, the treatment methods for EGVB are diverse. For stable patients after volume resuscitation, cautious transfusion is advised, with a transfusion threshold of 7 g/dL. For hemostatic treatment, vasoactive agents such as octreotide, somatostatin, or terlipressin, together with endoscopic interventions such as variceal ligation, should be used. A transjugular intrahepatic portosystemic shunt is used for resuscitation when uncontrolled bleeding occurs. It is advisable to administer prophylactic antibiotics, such as ceftriaxone, for 7 days or until the patient is discharged. For patients at high risk of recurrent bleeding, specifically those with a Child-Turcotte-Pugh B score between 8 and 9 points and active bleeding or a Child-Turcotte-Pugh C score ranging from 10 to 13 points, pre-emptive transjugular intrahepatic portosystemic shunt within 72 hours of admission could prolong their survival. For patients who are not at high risk, β-blockers combined with variceal ligation are recommended [1]. These conditions not only intensify patients’ pain but also shorten their lifespan and increase their economic burden. Thus, early identification of the risk of esophagogastric varices (EGV) in patients with LC is crucial for preventing EGVB, lowering its threat to health and life and the economic burden on families.

Endoscopy, which is interventional, is the primary tool for diagnosing esophageal variceal bleeding (EVB) and EGVB. As the gold standard for detecting EVB and EGVB, endoscopy is also recommended for diagnosing and screening for EGV and assessing the risk of bleeding [5]. Nonetheless, endoscopic examination is an invasive procedure, and a portion of patients may refuse this examination due to low tolerance. Moreover, repeated procedures might diminish long-term adherence. Furthermore, this examination may also induce EGVB in low-risk patients. Thus, recent studies have focused on the development of noninvasive or minimally invasive tools to identify the risks of EGV at an early stage. However, effective tools for the early prediction of EGV remain lacking. Recently, as machine learning (ML) has been increasingly used in medical diagnostics, some studies have investigated the feasibility of using ML techniques to evaluate the risk of EGVB in patients with LC. Nevertheless, their detection performance varies notably with the modeling approach or the variables used. Currently, there is insufficient evidence to demonstrate the feasibility of using ML to identify EVB and EGVB in patients with LC. Thus, this study included original studies that developed prediction models for esophageal or gastric variceal bleeding in patients with LC under different modeling variables. The aim of this study was to review the performance of ML in the early identification of EVB and EGVB in patients with LC, providing evidence for the advancement and updating of early risk assessment tools for EGV.

## Methods

### Study Registration

This study was performed based on the PRISMA (Preferred Reporting Items for Systematic Reviews and Meta-Analyses) guidelines (Checklist 1) and was prospectively registered with PROSPERO (ID CRD42024585100).

### Eligibility Criteria

Before screening the original studies, we established comprehensive eligibility criteria (Table 1).

**Table 1.** Eligibility criteria for screening the original studies for this systematic review.

Items	Inclusion criteria	Exclusion criteria
Population	Patients with LC^a^ as the study participants.	Studies that did not strictly distinguish between patients with liver cirrhosis and those with hepatocellular carcinoma.
Intervention	Studies that developed prediction models for the risk of EVB^b^ or EGVB^c^.	Studies that solely explored risk factors without creating a complete prediction model, studies that only centered on validated mature scales, studies on the predictive accuracy of a single factor for outcomes, and studies that did not strictly distinguish EVB or EGVB from other upper gastrointestinal bleeding.
Comparison	None	None
Outcome	Studies reporting any of the following outcome measures to evaluate the prediction model: c-index, sensitivity, specificity, accuracy, recall, precision, confusion matrix, *F*_1_-score, and calibration curve.	None
Study design	Studies adopting case-control, cohort, and cross-sectional designs; and original studies reported in English.	Conference abstracts that were published without peer review and for studies that repeatedly trained and validated models based on the same dataset, only the most recently published study was included.

a LC: liver cirrhosis.

b EVB: esophageal variceal bleeding

c EGVB: esophagogastric variceal bleeding.

### Data Sources and Search Strategy

PubMed, Web of Science, Embase, and the Cochrane Library were searched up to August 21, 2024. Both subject terms and free terms were used to design search terms. No restrictions regarding region or period were imposed. Multimedia Appendix 1 provides the detailed search strategy.

### Study Selection and Data Extraction

Articles retrieved from the databases were imported into EndNote (Clarivate Analytics). First, duplicates were removed. By reading the titles and abstracts, potentially eligible original studies were chosen. The full texts of these studies were then downloaded and read to determine eligible articles. Subsequently, a standardized spreadsheet was developed to extract data (eg, study title, first author, publication year, and study type) from the included articles. Two investigators independently screened the studies, extracted the data, and then cross-checked the results. Any disagreements were resolved through consultation with a third investigator.

### Risk of Bias Assessment

The risk of bias (ROB) in the included studies was appraised using the Prediction Model Risk of Bias Assessment Tool (PROBAST). This tool encompasses 4 domains: participant, predictor variable, outcome, and statistical analysis. Questions in each domain could be answered with 3 responses: yes or probably yes, no or probably no, and no information. A domain was deemed to have high ROB if any question was answered with no or probably no. A domain was deemed to have low ROB if all questions were answered with yes or probably yes. The overall ROB was low if all domains were considered low ROB. The overall ROB was high if at least one domain was considered to have a high ROB. Two investigators independently appraised the ROB using the PROBAST, and their results were cross-checked. Disagreements, if any, were resolved by a third investigator.

### Synthesis Methods

For evaluating the overall accuracy, a meta-analysis of the c-indices of ML models was performed. In certain original studies, the 95% CI and SEs for the c-index were missing. We referred to the study by Debray et al [6] to estimate the SEs. Heterogeneity across studies was assessed using the *I*^2^ statistic. A random-effects model was used if *I*^2^ was greater than 50%, whereas a fixed effects model was adopted if *I*^2^ was less than 50%. Additionally, the sensitivity and specificity, derived from the diagnostic quadrangle table, were pooled through bivariate mixed effects models. However, such a table was not available in many of the original studies. In this case, the number of cases with sensitivity, specificity, and precision was combined to compute the estimates. Sensitivity and specificity were extracted based on the optimal Youden index. This meta-analysis was performed in Stata (version 15.0; StataCorp LLC).

## Results

### Study Selection

**Figure 1.**
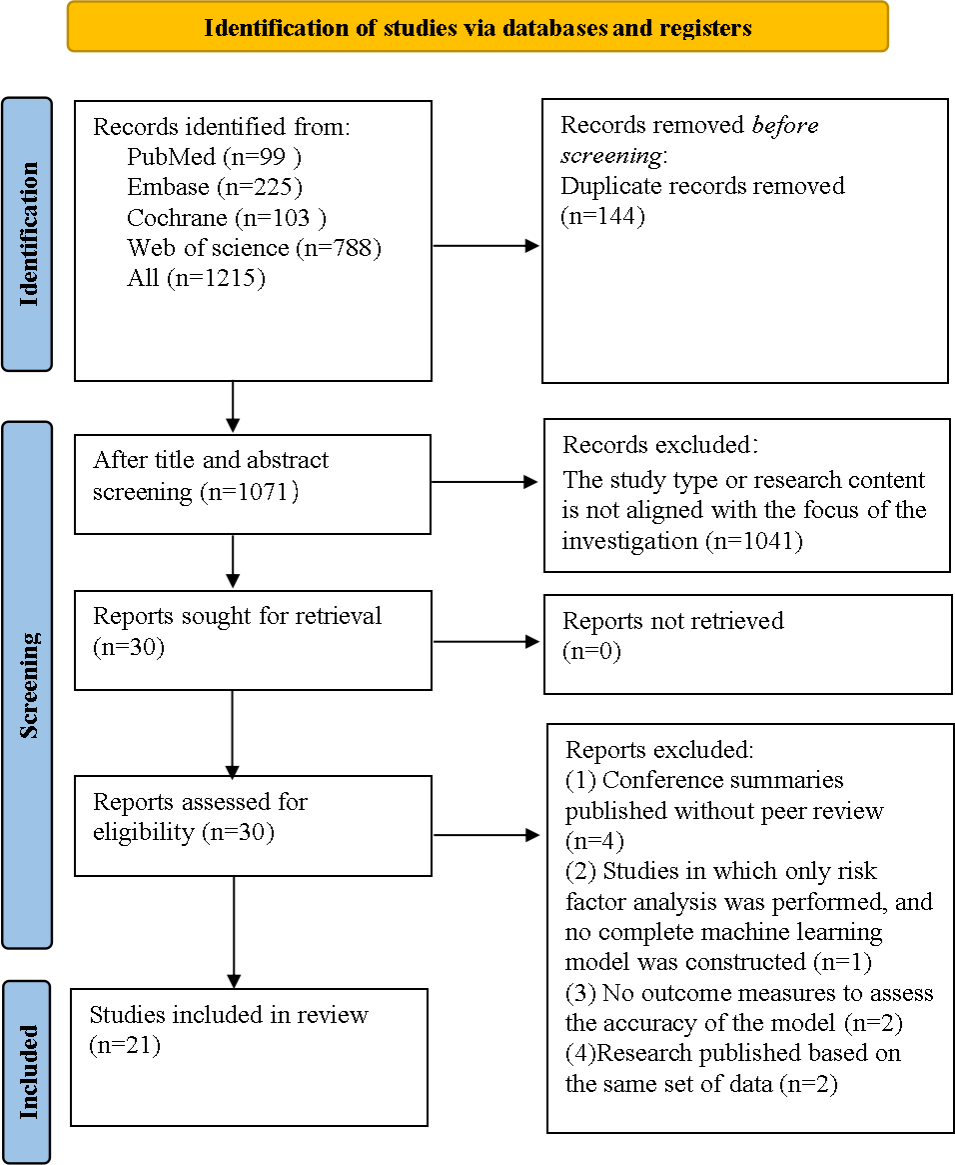
.PRISMA (Preferred Reporting Items for Systematic Reviews and Meta-Analyses) flowchart of the literature screening process.

In total, 1215 articles were retrieved from the databases. Following the removal of 144 (11.85%) duplicates, 1071 (88.15%) articles were left. In total, 1041(85.68%) articles were excluded due to unrelated topics, including meta-analyses, reviews, guidelines, animal experiments, case reports, letters, and registration protocols. The full texts of the remaining 30 (2.47%) articles were then downloaded and read. We further excluded 4 (0.33%) conference abstracts that had not undergone peer review, 1 (0.08%) study that analyzed only risk factors without developing a complete ML model, 2 (0.16%) studies without an accuracy assessment of model outcomes, and 2 (0.16%) studies published based on the same dataset of another 2 studies. Finally, 21 (1.73%) articles were included [7-27] (Figure 1).

### Study Characteristics

The included studies were published between 2014 and 2024, involving a total of 7011 patients with LC. Among these studies, 12 studies included 1412 cases of EVB [8,10-15,19-22,25], and 9 studies included 733 cases of EGVB [7-9,11,12,16,17,26,27]. These studies were conducted in 4 countries: 18 studies in China [7-20,23,25-27], 1 study in Egypt [21], 1 study in Iran [22], and 1 study in South Korea [24]. Among the included studies, 14 were case-control studies [7,12-16,18-20,22,24-27], 6 were cohort studies [8-11,21,23], and 1 was a cross-sectional study [15]. There were 16 single-center studies [7-9,12-17,19-23,25,27] and 5 multicenter studies [10,11,18,24,26]. Nine articles [7-9,11,12,16,17,26,27] centered on EGVB, and 12 articles focused on EVB [10,13-15,18-25]. Fourteen studies clearly outlined the method used to generate the validation set. Only 3 studies used external validation [10,18,19]. Two studies used time-based split [8,25], while the remaining studies conducted internal validation with random sampling. The models involved were mainly logistic regression models, while only a few studies constructed other types of models. Fifteen models were constructed based on clinical features [7-9,11,15-25]. Three models were based on radiomics [13,26,27]. Four models were based on radiomics and clinical features [12,14,26,27], and 1 model was based on endoscopic features in 1 study [10]. The detailed results are presented in Table 2.

**Table 2.** Basic characteristics of the included studies.

First author	Year of publication	Country of authors	Study type	Patient source	Predicted events	Event cases (n=2145), n (30.59%)	Cases (n=7011), n	Cases in training set (n=5554), n	Generation method of validation set	Cases in the validation set (n=1457), n	Model type	Modeling variables
Liu et al [7]	2022	China	Case-control study	Single center	EGVB^a^	77	330	220	Random sampling	110	LR^b^	Clinical features (emotional stimuli, improper diet, overwork, lower temperature, and increased abdominal pressure)
Zhang et al [8]	2022	China	Prospective cohort study	Single center	EGVB	52	172	128	Time-based split	44	Cox^c^	Clinical features (vPPG^d^, PLT^e^, albumin, and INR)^f^
Tan et al [9]	2023	China	Prospective study (cohort)	Single center	EGVB	74	230	160	Random sampling	70	LR	Clinical features (right liver lobe volume, diameters of maximum esophagogastric varices, and portal vein system)
Wang et al [10]	2023	China	Retrospective cohort study	Multicenter	EVB^g^	121	502	275	Random sampling and external validation	227	DL^h^, XGBoost^i^, GLM^j^, GBM^k^, and RF^l^	Endoscopic features (EV^m^ bleeding image)
Hou et al [11]	2023	China	Prospective cohort study	Multicenter	EGVB	131	1100	999	Random sampling	101	Cox	Clinical features (gender, alcohol and smoking history, decompensation, ascites, location and size of varicose veins, ALT^n^, GGT^o^, HCT^p^, and NLR^q^ levels, and RBC^r^ count)
Luo et al [12]	2023	China	Retrospective case-control study	Single center	EGVB	88	211	149	Random sampling	62	LR	Radiomics and clinical features (albumin, fibrinogen, portal vein thrombosis, aspartate aminotransferase, and spleen thickness)
Meng et al [13]	2021	China	Retrospective case-control study	Single center	EVB	39	173	121	Random sampling	52	Cox	Radiomics
Liu et al [14]	2022	China	Retrospective case-control study	Single center	EVB	69	317	222	Random sampling	95	LR	Radiomics and clinical features (hemoglobin)
Xu et al [15]	2024	China	Case-control study	Single center	EVB	114	241	160	Random sampling	81	LR	Clinical features (spleen length and platelet distribution width PDW^s^)
Lin et al [17]	2024	China	Retrospective cross-sectional study	Single center	EGVB	70	210	210	None^t^		LR	Clinical features (LSM^u^ and GBS^v^ scoring)
Zhang et al [18]	2023	China	Retrospective analysis	Multicenter	EVB	77	714	527	External validation	187	Cox	Clinical features (the size of varices, red wale marks, ascites, spleen thickness, γ-glutamyltransferase, and hematocrit)
Yang et al [19]	2023	China	Retrospective case-control study	Single center	EVB	98	200	140	External validation	60	LR	Clinical features (SSM^w^ and LSM)
Fu et al [20]	2023	China	Case-control study	Single center	EVB	40	78	78	None		LR	Clinical features (age, gender, platelet count, Child-Pugh classification, and normalized iodine density)
Samy et al [21]	2022	Egypt	Prospective cohort study	Single center	EVB	42	85	85	None		LR	Clinical features (main coronary vein size, para-esophageal collateral, short gastric collateral, gastric varicosities, child class C, and size of EVs >4 mm)
Salahshour et al [22]	2020	Iran	Retrospective case-control study	Single center	EVB	50	124	124	None		LR	Clinical features (presence of SGC^x^, SGC size, presence of EV, and MELD^y^ score)
Li et al [16]	2022	China	Retrospective case-control study	Single center	EGVB	85	142	142	None		LR	Clinical features (spleen volume expansion rate, serum sodium level, hemoglobin level, and prothrombin time)
Xu et al [23]	2014	China	Prospective cohort study	Single center	EVB	51	416	416	None		LR	Clinical features (ΔMELD, LGVV^z^, and LGVBFD)^aa^
Heo et al [24]	2019	Korea	Retrospective case-control study	Multicenter	EVB	61	262	262	None		LR	Clinical features (ARFI^ab^ velocity, spleen diameter, platelet count, ASPS^ac^, Child-Pugh class, EV status, portal hypertensive gastropathy, etiology of cirrhosis, age)
Liu et al [25]	2022	China	Retrospective case-control study	Single center	EVB	650	1099	823	Time-based split	276	LR	Clinical features (PLT, Hb^ad^, ALB^ae^/GLB^af^, FBG^ag^, Cl^ah^, and CTPD^ai^)
Yang et al [26]	2019	China	Retrospective case-control study	Multicenter	EGVB	91	295	236	Random sampling	59	RF	Radiomics and clinical features (sex, portal vein thrombosis, and portal hypertension)
Zhang et al [27]	2023	China	Retrospective study	Single center	EGVB	65	110	77	Random sampling	33	LR	Radiomics and clinical features (PLT)

a EGVB: esophagogastric variceal bleeding.

b LR: logistic regression.

c Cox: Cox regression.

d vPPG: virtual portal pressure gradient.

e PLT: platelet.

f INR: international normalized ratio.

g EVB: esophageal variceal bleeding.

h DL: deep learning.

i XGBoost: Extreme Gradient Boosting.

j GLM: general linear regression.

k GBM: gradient boost machine.

l RF: random forest.

m EV: esophageal variceal.

n ALT: alanine aminotransferase.

o GGT: γ-glutamyl transferase.

p HCT: hematocrit.

q NLR: neutrophil-lymphocyte ratio.

r RBC: red blood cell.

s PDW: platelet distribution width.

t In the generation method of the validation set, “None” indicates that there is no independent validation set.

u LSM: liver stiffness measurement.

v GBS: Glasgow-Blatchford score.

w SSM: spleen stiffness measurement.

x SGC: short gastric.

y MELD: Model for End-stage Liver Disease.

z LGVV: left gastric vein blood flow velocity.

aa LGVBFD: left gastric vein blood flow direction.

ab ARFI: acoustic radiation force impulse.

ac ASPS: ARFI–spleen diameter-to-platelet ratio scores.

ad Hb: hemoglobin.

ae ALB: albumin.

af GLB: globulin.

ag FBG: fasting blood glucose.

ah Cl: serum chloride.

ai CTPD: computed tomography portal vein diameter.

### ROB Assessment

The ROB in the included models was assessed. Fourteen models were from case-control studies, introducing a high ROB in participant selection. Fifteen models were built based on clinical features, resulting in a higher ROB in the predictive factors. Three models were based on radiomics, and thus, it was challenging to assess ROB. In terms of results, the included studies clearly outlined diagnostic criteria for EVB and EGVB, and no evidence of high ROB was noted in these diagnostic standards.

**Figure 2.**
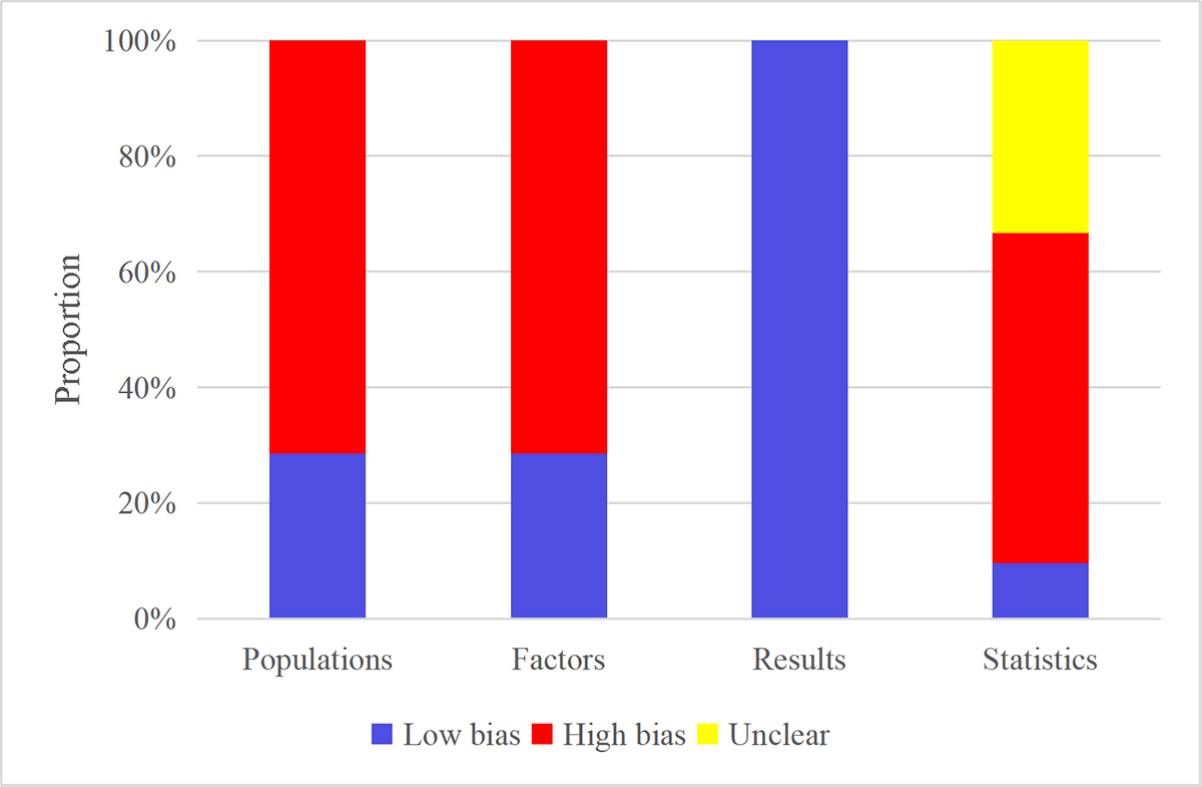
Risk of bias assessment results of the included studies by the Prediction Model Risk of Bias Assessment Tool (PROBAST).

Of the models included, the number of cases was small in 9 training sets, resulting in a higher ROB. For 7 models, the number of cases in the validation set could not be estimated, leading to an unclear ROB. Regarding the handling of missing values, missing values were deleted in 6 models, leading to a high ROB. Only one study applied univariate analysis to select variables for the models, and 7 studies did not provide details on their variable selection methods. Among the included models, only one model was cross-validated, and thus, a high ROB was identified in the study. The full assessment results are available in Figure 2.

### Meta-Analysis

#### Esophageal Variceal Bleeding

##### Pooled Results

**Figure 3.**
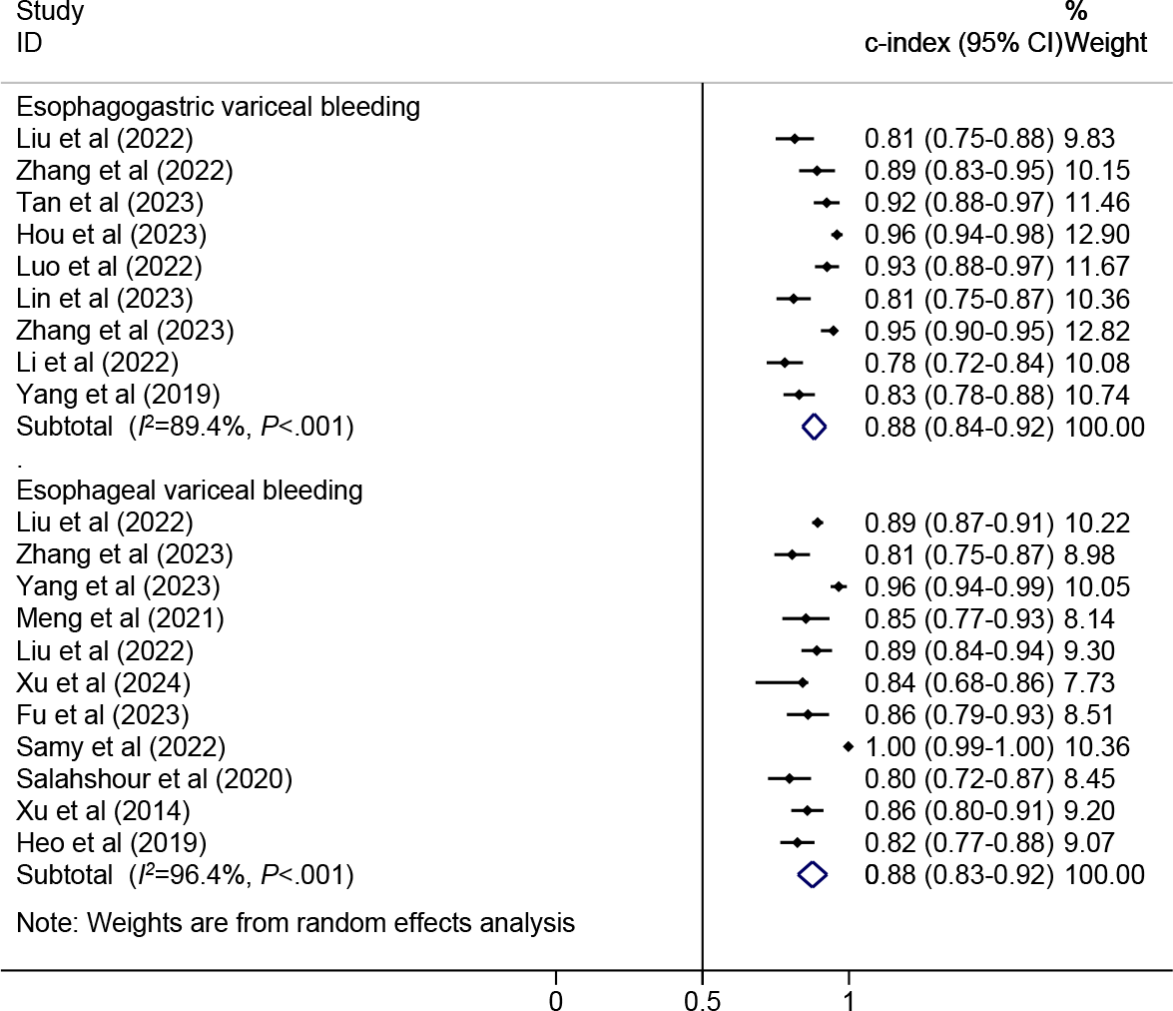
Forest plot for meta-analysis of c-index of machine learning for predicting esophageal variceal bleeding and esophagogastric variceal bleeding in patients with liver cirrhosis in the training set [7-9,11-24,26,27].

Twelve models were created for predicting EVB. The meta-analysis was performed using a random-effects model. The pooled c-index, sensitivity, and specificity in the training set were 0.88 (95% CI 0.83‐0.92), 0.93 (95% CI 0.83‐0.97), and 0.75 (95% CI 0.65‐0.84), respectively (Figures3  4). The pooled c-index, sensitivity, and specificity in the validation set were 0.85 (95% CI 0.77‐0.92), 0.93 (95% CI 0.87‐0.96), and 0.66 (95% CI 0.46‐0.82), respectively (Figures5  6).

**Figure 4.**
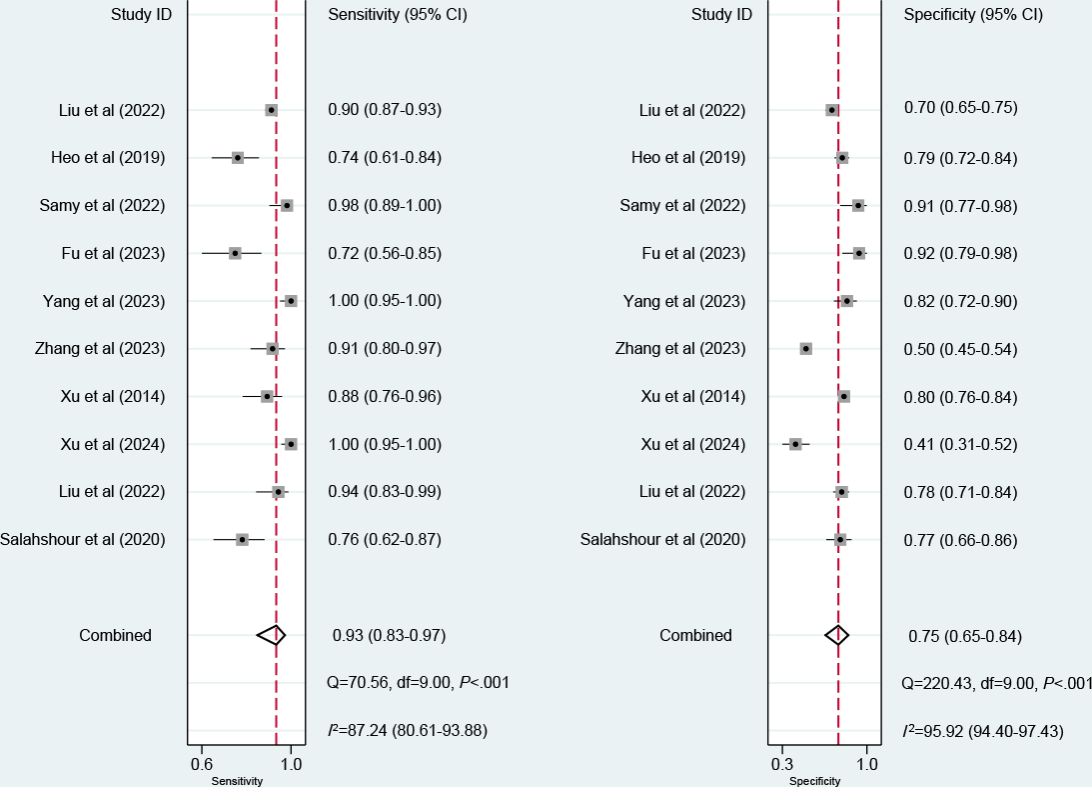
Forest plot for meta-analysis of sensitivity and specificity of machine learning for predicting esophageal variceal bleeding in patients with liver cirrhosis in the training set [14,15,18-25].

**Figure 5.**
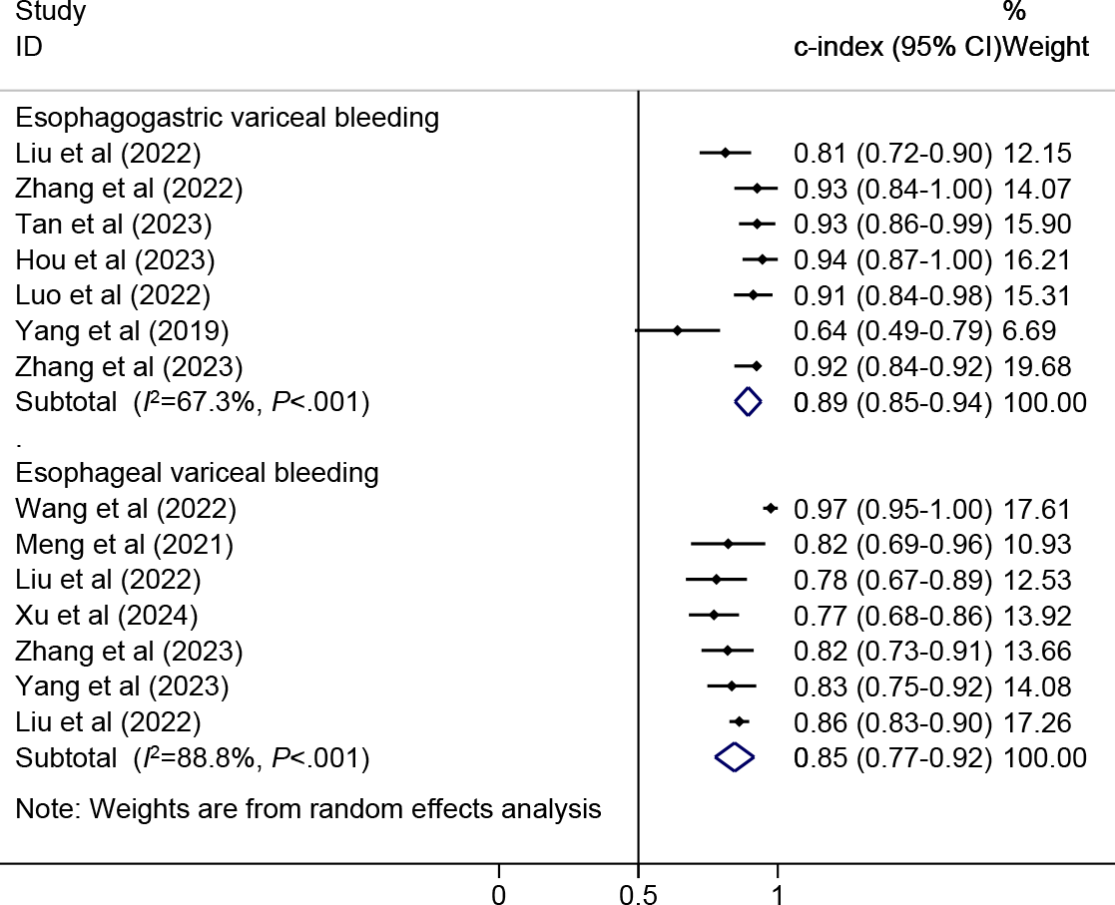
Forest plot for meta-analysis of c-index of machine learning for predicting esophageal variceal bleeding and esophagogastric variceal bleeding in patients with liver cirrhosis in the validation set [7-15,18,19,25-27].

**Figure 6.**
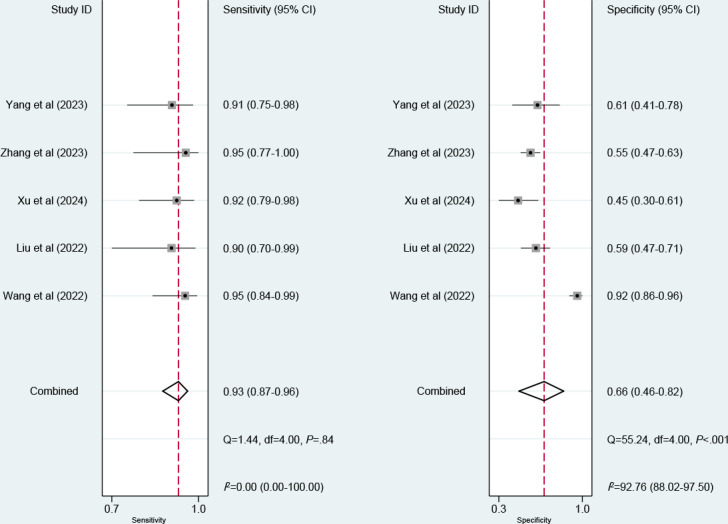
Forest plot for meta-analysis of sensitivity and specificity of machine learning for predicting esophageal variceal bleeding in patients with liver cirrhosis in the validation set [10,14,15,18,19].

##### Subgroup Analysis

A subgroup analysis was performed based on modeling variables and the generation method of the validation set. In the subgroup analysis by model variables, models were grouped based on clinical features, radiomics-based models, models based on radiomics and clinical features, and models based on endoscopic features. In the training set, the c-index was 0.88 (95% CI 0.82‐0.93) for models based on clinical features, 0.85 (95% CI 0.77‐0.93) for radiomics-based models, and 0.89 (95% CI 0.84‐0.94) for models based on radiomics and clinical features (Multimedia Appendix 2). In the validation set, the c-index was 0.84 (95% CI 0.80‐0.88) for models based on clinical features, 0.82 (95% CI 0.69‐0.96) for radiomics-based models, 0.78 (95% CI 0.67‐0.89) for models based on radiomics and clinical features, and 0.97 (95% CI 0.95‐1.00) for models based on endoscopic features (Multimedia Appendix 3). In the subgroup analysis by the generation method of the validation set, models were grouped as random sampling, external validation, and time-based split. The c-index was 0.78 (95% CI 0.72‐0.85) for models based on random sampling, 0.88 (95% CI 0.77‐1.00) for external validation, and 0.86 (95% CI 0.83‐0.90) for time-based split (Multimedia Appendix 4).

### Esophagogastric Variceal Bleeding

#### Pooled Results

Nine models were created for predicting EGVB. The meta-analysis was conducted using the random-effects model. The pooled results revealed that the c-index, sensitivity, and specificity in the training set were 0.88 (95% CI 0.84‐0.92), 0.90 (95% CI 0.78‐0.96), and 0.79 (95% CI 0.58‐0.91), respectively (Figures3  7). The pooled c-index, sensitivity, and specificity in the validation set were 0.89 (95% CI 0.85‐0.94), 0.77 (95% CI 0.66‐0.85), and 0.81 (95% CI 0.67‐0.90), respectively (Figures5  8).

**Figure 7.**
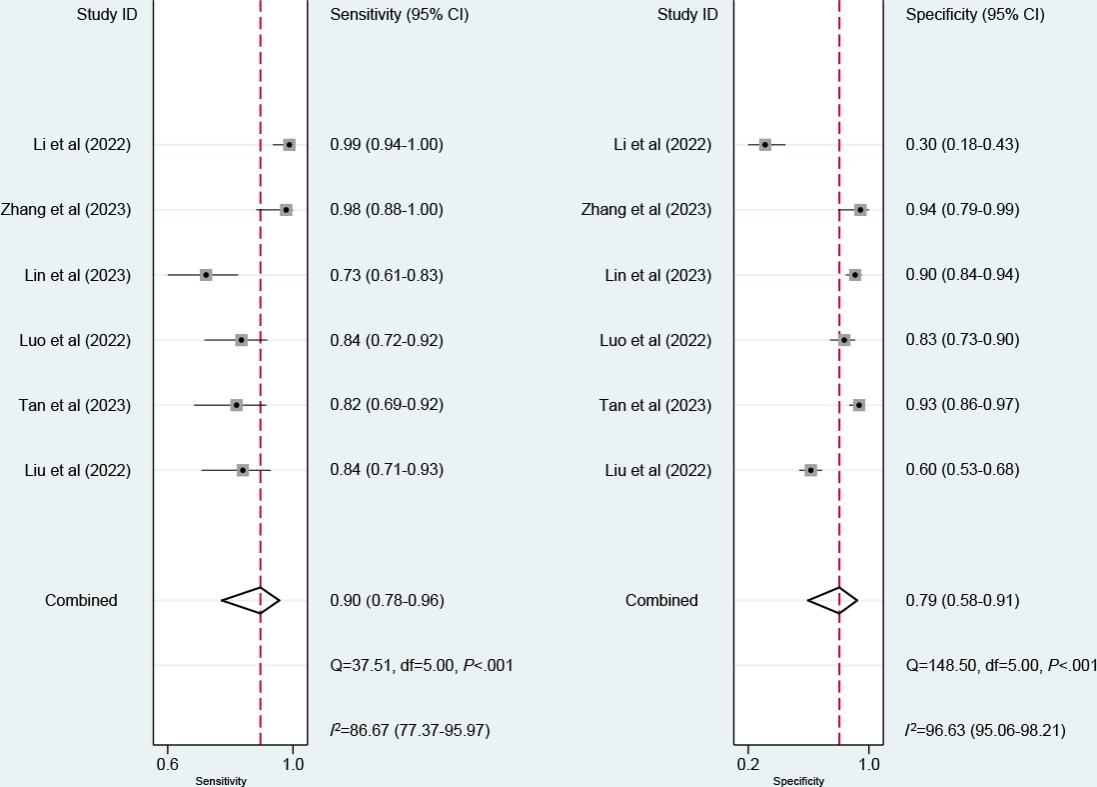
Forest plot for meta-analysis of sensitivity and specificity of machine learning for predicting esophagogastric variceal bleeding in patients with liver cirrhosis in the training set [7,9,12,16,17,27].

**Figure 8.**
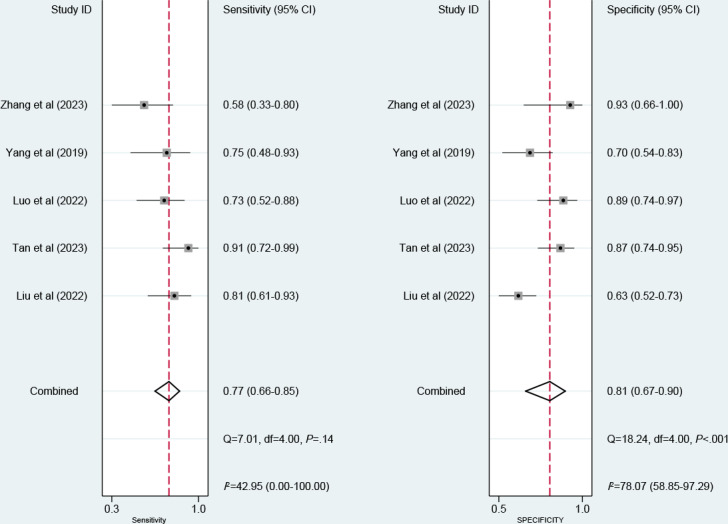
Forest plot for meta-analysis of sensitivity and specificity of machine learning for predicting esophagogastric variceal bleeding in patients with liver cirrhosis in the validation set [7,9,12,26,27].

#### Subgroup Analysis

A subgroup analysis was performed based on modeling variables and the generation method of the validation set. In the subgroup analysis by model variables, models were grouped as models based on clinical features and models based on radiomics and clinical features. In the training set, the c-index was 0.87 (95% CI 0.80‐0.93) for models based on clinical features and 0.90 (95% CI 0.84‐0.97) for models based on radiomics and clinical features (Multimedia Appendix 5). In the validation set, the c-index was 0.91 (95% CI 0.86‐0.96) for models based on clinical features, 0.85 (95% CI 0.75‐0.96) for models based on radiomics and clinical features (Multimedia Appendix 6). In the subgroup analysis by the generation method of the validation set, models were grouped as random sampling and time-based split. The c-index was 0.89 (95% CI 0.83‐0.94) for models based on random sampling and 0.93 (95% CI 0.85‐1.00) for time-based split (Multimedia Appendix 7).

## Discussion

### Principal Findings

The risk of EVB and EGVB in patients with LC is high. Our study demonstrates that ML may be a feasible method for the early prediction of the risk of EVB and EGVB. In the validation set, the c-index, sensitivity, and specificity for ML models in the prediction of EVB were 0.85 (95% CI 0.77‐0.92), 0.93 (95% CI 0.87‐0.96), and 0.66 (95% CI 0.46‐0.82), respectively. Moreover, in the validation set, the c-index, sensitivity, and specificity for ML models in the prediction of EGVB were 0.89 (95% CI 0.85‐0.94), 0.77 (95% CI 0.66‐0.85), and 0.81 (95% CI 0.67‐0.90), respectively. Notably, 2 studies by Yang and Xu reported a sensitivity of 1.00 (100%) in the training set, but their specificity did not reach 1.00. Therefore, no perfect separation was observed. Furthermore, we also found that in the validation set, both studies reported sensitivities greater than 0.9 in the validation set, indicating no excessive overfitting. Therefore, this result indicates that the c-index and sensitivity of ML models are relatively high.

### Comparison With Previous Studies

In patients with LC, EVB and EGVB represent a noticeable challenge that must be addressed in clinical research. Previous studies have established prediction models for EVB and EGVB. For instance, Malik et al [28] explored the use of ML for EVB or grading in patients with LC. Their results indicate that ML is highly promising for predicting esophageal varices in patients with LC and may reduce the necessity for minor surgeries. Nevertheless, their study points out that there is considerable heterogeneity and methodological limitations in their included studies, resulting in a high or unclear ROB. In the future, large-scale prospective trials are needed, and the evaluation standards for ML should be standardized to improve the practicality of these models in clinical settings.

Radiomics has recently attracted increasing attention in research on LC. In the study by Xue et al [29], transfer learning (TL) radiomics using multimodal ultrasound imaging was used for classifying the stage of liver fibrosis (LF). Their results revealed that in both the gray scale modality (GM) and elastogram modality (EM), TL exhibits superior diagnostic performance to non-TL approaches, with significantly higher areas under the receiver operating characteristic curve (AUCs; all *P*<.01). Single-modal GM and EM both outperform liver stiffness measurement (LSM) and serum indices for staging LF (all *P*<.001). The prediction performance of multimodal GM+EM is higher than GM+LSM, GM and EM alone, LSM, and biomarkers (all *P*<.05). The AUC of multimodal GM+EM for staging LF is 0.950 for S4, 0.932 for S3 or more, and 0.930 for S2 or more. Yan et al [30] used multiphase magnetic resonance imaging–based radiomics to forecast the histological grading of HCC. According to their results, 33.8% of the cases were high-grade HCCs. The levels of α-fetoprotein (odds ratio 1.89) and tumor size (>5 cm; odds ratio 2.33) are strongly associated with HCC grade. The performance of the combined model is high in predicting the grade of HCC in the test dataset (AUC=0.801), with favorable calibration and clinical utility. Park et al [31] reviewed the applications of radiomics and deep learning (DL) in liver diseases. According to their results, radiomics and DL are promising techniques for imaging-based assessment of liver diseases. Recent studies have reported the potential uses of radiomics and DL in staging LF, detecting portal hypertension, describing focal liver lesions, and predicting malignant liver tumors. Consequently, radiomics has been receiving growing attention in the clinical diagnosis and treatment of LC.

In our study, the included variables primarily consisted of interpretable clinical features and radiomic features. The accuracy of radiomics-based models for predicting EVB and EGVB in patients with LC is relatively favorable. In the included studies, 5 models were constructed based on radiomics features [12-14,26,27], among which 1 model was based on radiomics features [13] and 4 models were based on radiomics and clinical features [12,14,26,27]. The 5 studies describe the detection of bleeding in LC based on computed tomography (CT) or magnetic resonance imaging scans. Luo et al [12] developed a model based on 5 CT features from the liver and 3 CT features from the spleen. Its AUCs were 0.817 and 0.741 in the training and validation cohorts, respectively. Moreover, the clinical-radiomics model also exhibits notably predictive performance, with an AUC of 0.925 in the training cohort and 0.912 in the validation cohort. In the study by Meng et al [13], the Rad-score Liver-Spleen, based on 10 features, demonstrated strong discriminative performance. Its c-index is 0.853 (95% CI 0.776‐0.904) in the training cohort and 0.822 (95% CI 0.749‐0.875) in the validation cohort. According to Liu et al [14], the AUCs of their model are 0.89 (95% CI 0.84‐0.94) and 0.78 (95% CI 0.68‐0.87) in the training and validation datasets, respectively. These studies demonstrate that radiomics-based ML models have high predictive accuracy.

Although only a few studies on radiomic features were included in this study, their findings reveal that radiomics is essential in the diagnosis and treatment of LC. Given the small number of studies, their effect sizes were not pooled. However, the c-index of radiomics-based models was examined in the training and validation sets for both EVB and EGVB. For EVB, the c-index values were 0.85 (95% CI 0.77‐0.93) and 0.82 (95% CI 0.69‐0.96) for radiomics-based models in the training and validation sets, respectively. For EGVB, the c-index values were 0.90 (95% CI 0.84‐0.97) and 0.85 (95% CI 0.75‐0.96) for models based on radiomics and clinical features in the training and validation sets, respectively. The data indicate that radiomics appears to have a favorable performance for early prediction of EVB and EGVB. Therefore, future studies should focus on applying radiomics for the early differentiation of EVB and EGVB. However, due to the limited number of studies on radiomics, the results should be interpreted with caution. The detection performance of models constructed with traditional clinical features appears to be similar to that of models based on radiomics features. However, due to the limited number of studies on radiomics, more radiomics methods need to be explored to assess the risk of EVB and EGVB in patients with LC. Therefore, more studies need to be included in future research.

Furthermore, there was only one study based on endoscopic features. Endoscopy is broadly used in the management of EGV in patients with LC. The European Society of Gastrointestinal Endoscopy Guideline [32] offers a comprehensive overview of the role of endoscopy in the diagnosis and treatment of variceal bleeding. However, given the limited number of studies based on endoscopic images, it is impossible to assess their performance. Overall, this study demonstrates that ML has high predictive accuracy. Nonetheless, a high ROB is observed. Moreover, it is difficult to assess the ROB of models based on endoscopic features. Therefore, we believe that ML is a feasible approach to predicting EVB and EGVB in patients with LC, but it comes with a high ROB.

We conducted subgroup analyses by modeling variables, including those based on clinical characteristics and those based on imaging, to further elucidate the sources of heterogeneity. However, subgroup analyses revealed substantial heterogeneity within the subgroups. Due to the limited number of included articles, we were unable to conduct subgroup analysis by additional variables. Furthermore, subgroup analysis remains a significant challenge for meta-analyses on ML models. Given the limited number of included articles, the interpretation of differences in results across different types of ML is limited. Heterogeneity between different model types is a concern in meta-analyses of ML models. Even within the same model, differences in training rules can contribute to heterogeneity. Furthermore, the number of cases remains a potential influencing factor. Overall, this study aimed to quantitatively summarize ML models for the early prediction of esophageal or gastric variceal bleeding in LC, better demonstrating the application value of ML. However, high heterogeneity was observed. Although we attempted to address the heterogeneity from multiple perspectives, it was difficult to further quantify the impact of these differences on the results due to population differences, varying methods of assessing bleeding, and a limited number of studies. Therefore, this may impose certain limitations on the interpretation of the results. Future research should further consider the impact of these factors on the results.

This study reveals a clear association between high AUC values and high ROB. Although several studies reported extremely high discrimination accuracy (AUC >0.90), PROBAST assessment showed that these studies generally had a high or unclear ROB. In the field of ML, near-perfect discrimination performance (AUC≈1.0) in small-sample, single-center studies is often a warning sign of methodological flaws rather than reflecting true performance. Specifically, among models with AUC ≥0.95, most were derived from single-center studies with a sample size of fewer than 200. These small sample studies with high AUC values mainly had the following problems in the PROBAST assessment: (1) the high ROB in participant selection was attributable to a retrospective design and case-control design; (2) the high ROB in analysis may be explained by the lack of appropriate validation strategy and treatment measures for overfitting; and (3) high ROB in predictors was due to insufficient transparency in variable selection and treatment. This suggests that limitations in methodology may lead to overestimation of model performance. Especially, extremely high AUC values observed in small-sample studies may reflect overfitting of the model to the training data rather than true clinical utility. This phenomenon may be due to multiple factors, such as occasional outperformance due to insufficient sample size, overfitting due to inadequate validation, and definitions of predictors and outcomes. The high ROB for the analysis domain in the PROBAST assessment (particularly the lack of measures to address overfitting) supports this explanation. Models with a c-index or AUC above 0.95 may be due to a small sample size and single-center design, with a potential risk of overfitting. Notably, high discrimination was also observed in larger, more rigorously designed multicenter trials [10].

These findings have important implications for clinical translation. First, for exceptionally high-performing models, the methodological rigor should be rigorously assessed. Second, future studies should include a sufficient sample size, perform cross-validation or external validation, and report calibration performance rather than focusing solely on discrimination. Finally, multicenter collaborations and prospective designs are crucial for generating generalizable predictive models. The discrimination performance of radiomics-based models in this study was high (AUC 0.91‐0.93). However, given the limited number of related studies (n=5) with small sample sizes and insufficient validation, these results should be interpreted with caution.

### Strengths and Limitations

This study is the first to explore ML in predicting the risk of EVB and EGVB in patients with LC, offering a strong reference for the future development or updating of intelligent tools. Nonetheless, certain limitations must be considered. First, owing to the small number of radiomics studies, this study only summarized the c-index of radiomics-based models and did not discuss their sensitivities and specificities. Hence, the results should be interpreted with caution. Second, only some of the studies explicitly described their validation sets, while others did not perform any model validation. Third, the models were primarily developed using internal validation methods, with no independent external validation, which, to some extent, restricts the interpretation of the results. Fourth, while current ML techniques demonstrate excellent sensitivity in validation datasets, the false-positive rate remains high. Future research should not only improve sensitivity but also optimize specificity. Fifth, given significant methodological and clinical heterogeneity in the current evidence, our results merely summarize the current state of application of ML. It has the potential to serve as an early predictive tool for esophageal or gastric variceal bleeding in LC. Future research should further improve the methodology and model performance to establish definitive clinical benchmarks. Sixth, although we conducted subgroup analysis based on the generation method of the validation set, there are few studies on external validation. The results of external validation need to be interpreted carefully. Seventh, there was excessive heterogeneity in the meta-analysis of sensitivity and specificity. Hence, the pooled estimates should be considered descriptive averages rather than true predictive performance. The results need to be interpreted with caution. Seventh, 18 of the 21 included studies were from China, which may limit the generalizability of the results to other regions. Therefore, the generalizability of the results still needs to be validated by more data. Finally, although our results show high AUC and c-index values, a large number of the included studies have a high ROB (particularly in the analysis domain, eg, small sample size, a lack of external validation, and potential overfitting). Hence, our results should be interpreted with caution.

### Conclusions

The performance of ML-based models for predicting EVB and EGVB in patients with LC is relatively favorable, particularly those based on radiomics. However, as the number of the included original studies is limited, it is impossible to explore the impact of validation set generation methods, model types, and other factors on outcomes. Therefore, future studies should include multicenter cohorts and integrate multi-omics databases to develop ML prediction tools with improved predictive performance and broader applicability, thereby developing specialized prevention strategies to reduce the risk of EVB and EGVB in patients with LC.

## Supplementary material

10.2196/78203 Multimedia Appendix 1 Literature search strategy.

10.2196/78203 Multimedia Appendix 2 Forest plot of meta-analysis of c-index of machine learning models based on different modeling variables for predicting esophageal variceal bleeding in patients with liver cirrhosis in the training set.

10.2196/78203 Multimedia Appendix 3 Forest plot of meta-analysis of c-index of machine learning models based on different modeling variables for predicting esophageal variceal bleeding in patients with liver cirrhosis in the validation set.

10.2196/78203 Multimedia Appendix 4 Forest plot of meta-analysis of c-index of machine learning models based on different methods of generating the validation set for predicting esophageal variceal bleeding patients with liver cirrhosis in the validation set.

10.2196/78203 Multimedia Appendix 5 Forest plot of meta-analysis of c-index of machine learning models based on different modeling variables for predicting esophagogastric variceal bleeding patients with liver cirrhosis in the training set.

10.2196/78203 Multimedia Appendix 6 Forest plot of meta-analysis of c-index of machine learning models based on different modeling variables for predicting esophagogastric variceal bleeding patients with liver cirrhosis in the validation set.

10.2196/78203 Multimedia Appendix 7 Forest plot of meta-analysis of c-index of machine learning models based on different methods of generating the validation set for predicting esophagogastric variceal bleeding patients with liver cirrhosis in the validation set.

10.2196/78203 Checklist 1 PRISMA checklist.

## Abbreviations

AUC: area under the receiver operating characteristic curve
CT: computed tomography
DL: deep learning
EGVB: esophagogastric variceal bleeding
EM: elastogram modality
EVB: esophageal variceal bleeding
GM: gray scale modality
HCC: hepatocellular carcinoma
LC: liver cirrhosis
LF: liver fibrosis
LSM: liver stiffness measurement
ML: machine learning
PRISMA: Preferred Reporting Items for Systematic Reviews and Meta-Analyses
PROBAST: Prediction Model Risk of Bias Assessment Tool
ROB: risk of bias
TL: transfer learning
