# Supplementary figures and images for: Machine Learning in Predicting the Risk of Esophagogastric Variceal Bleeding Among Patients With Liver Cirrhosis: Systematic Review and Meta-Analysis
Source: J Med Internet Res. 2026 Apr 8;28:e78203. doi: 10.2196/78203 (PMC13061106; doi:10.2196/78203)

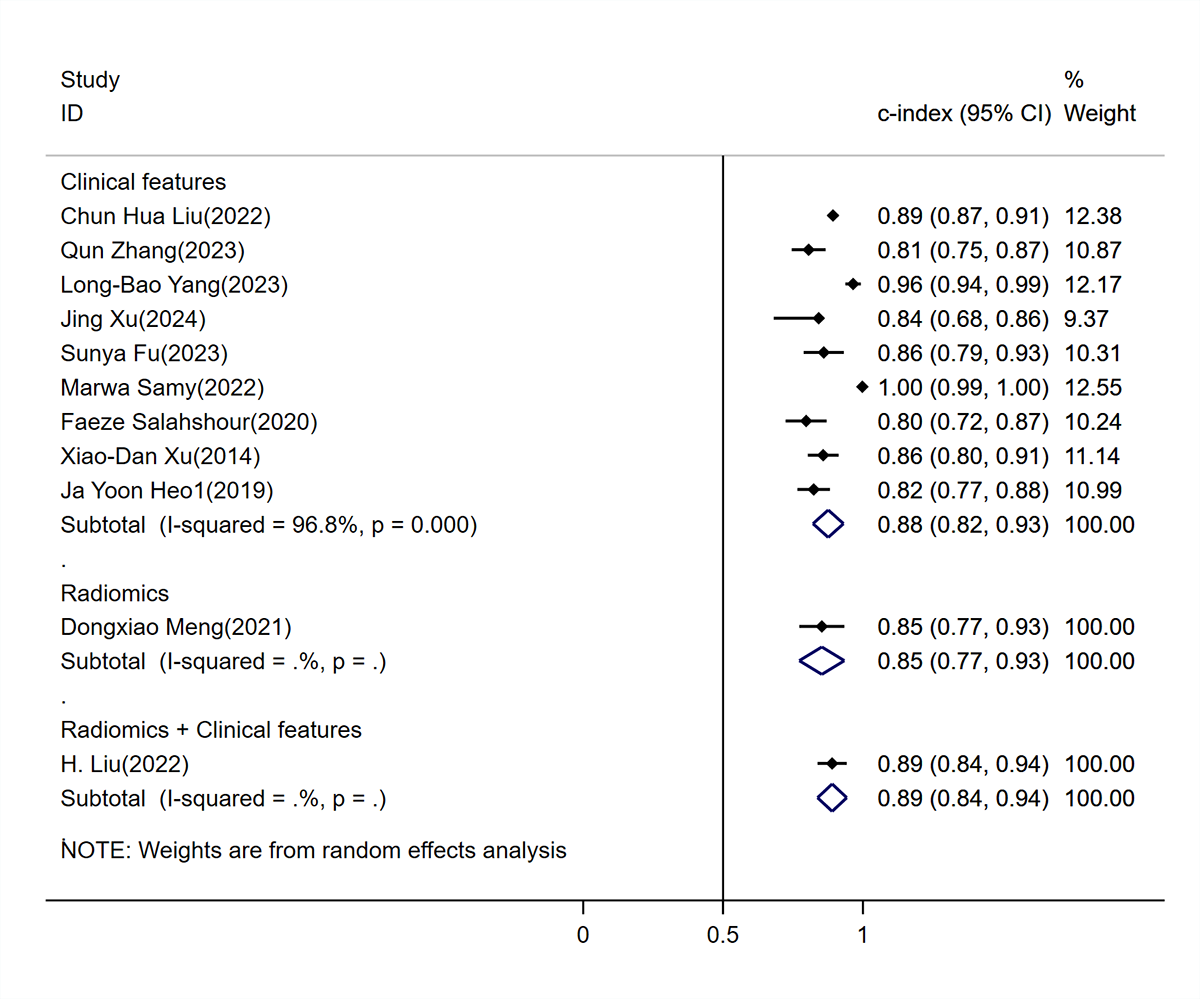

Supplement: Multimedia Appendix 1 [file jmir-v28-e78203-s001.png]

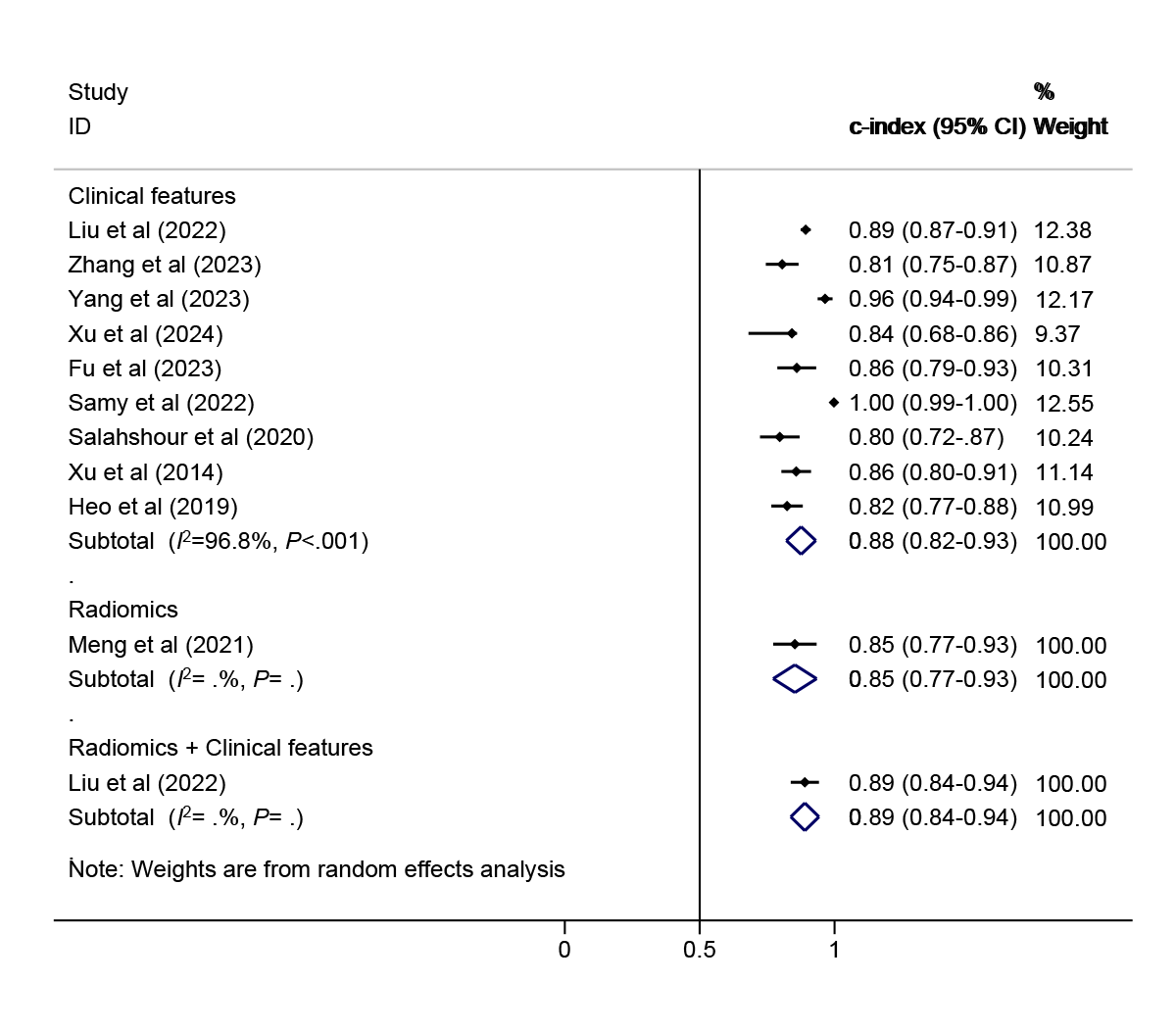

Supplement: Multimedia Appendix 2 [file jmir-v28-e78203-s002.png]

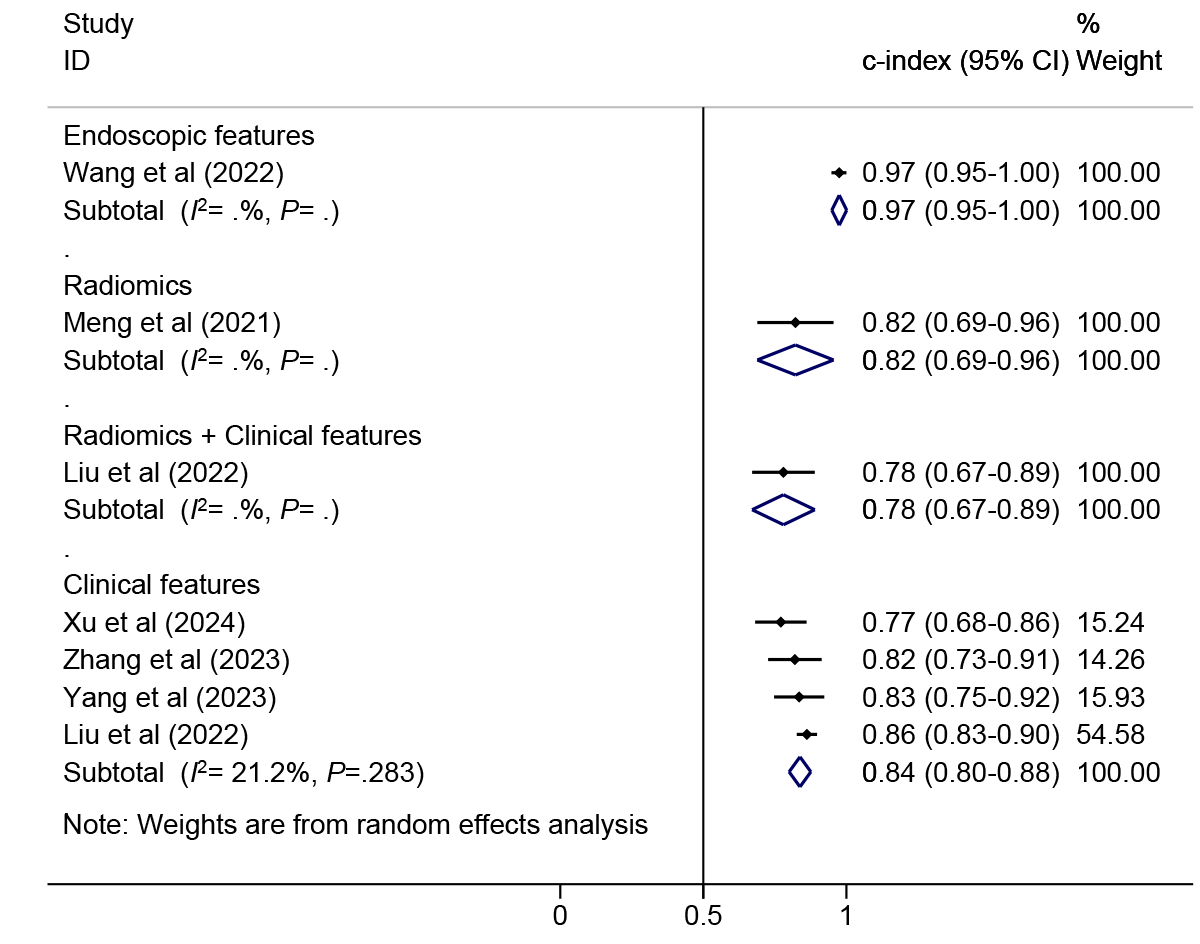

Supplement: Multimedia Appendix 3 [file jmir-v28-e78203-s003.png]

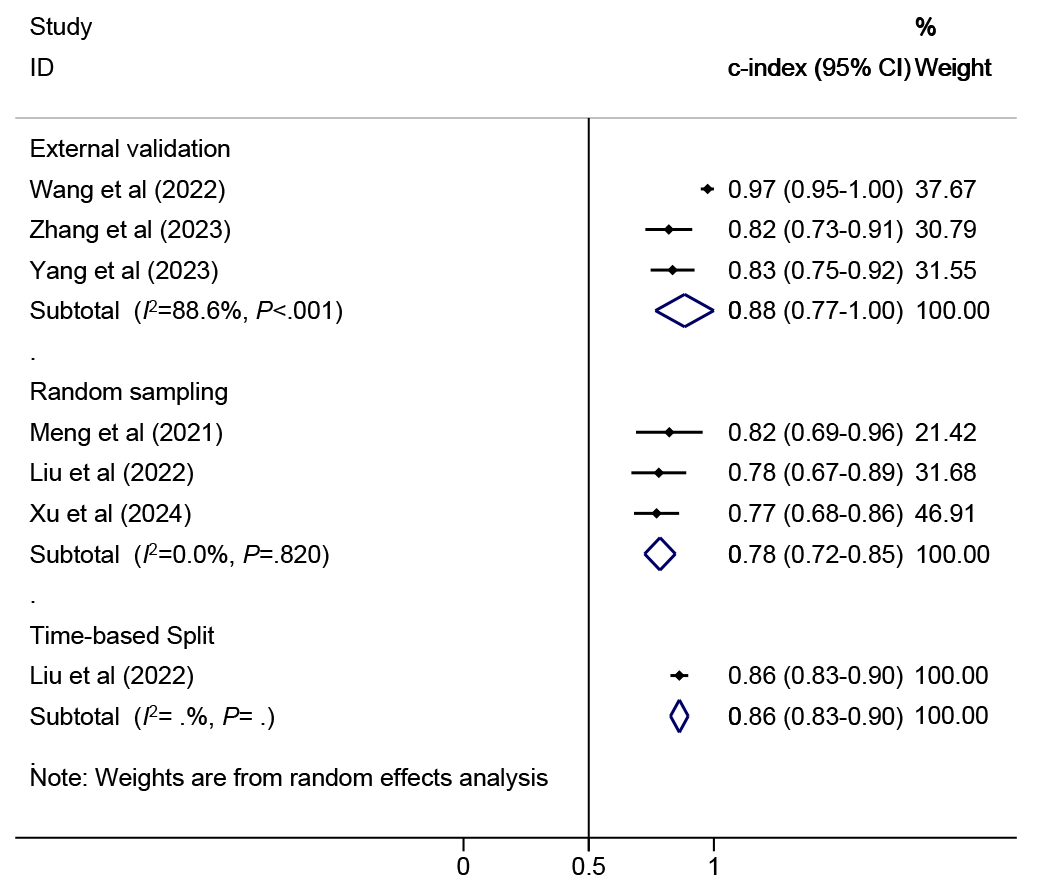

Supplement: Multimedia Appendix 4 [file jmir-v28-e78203-s004.png]

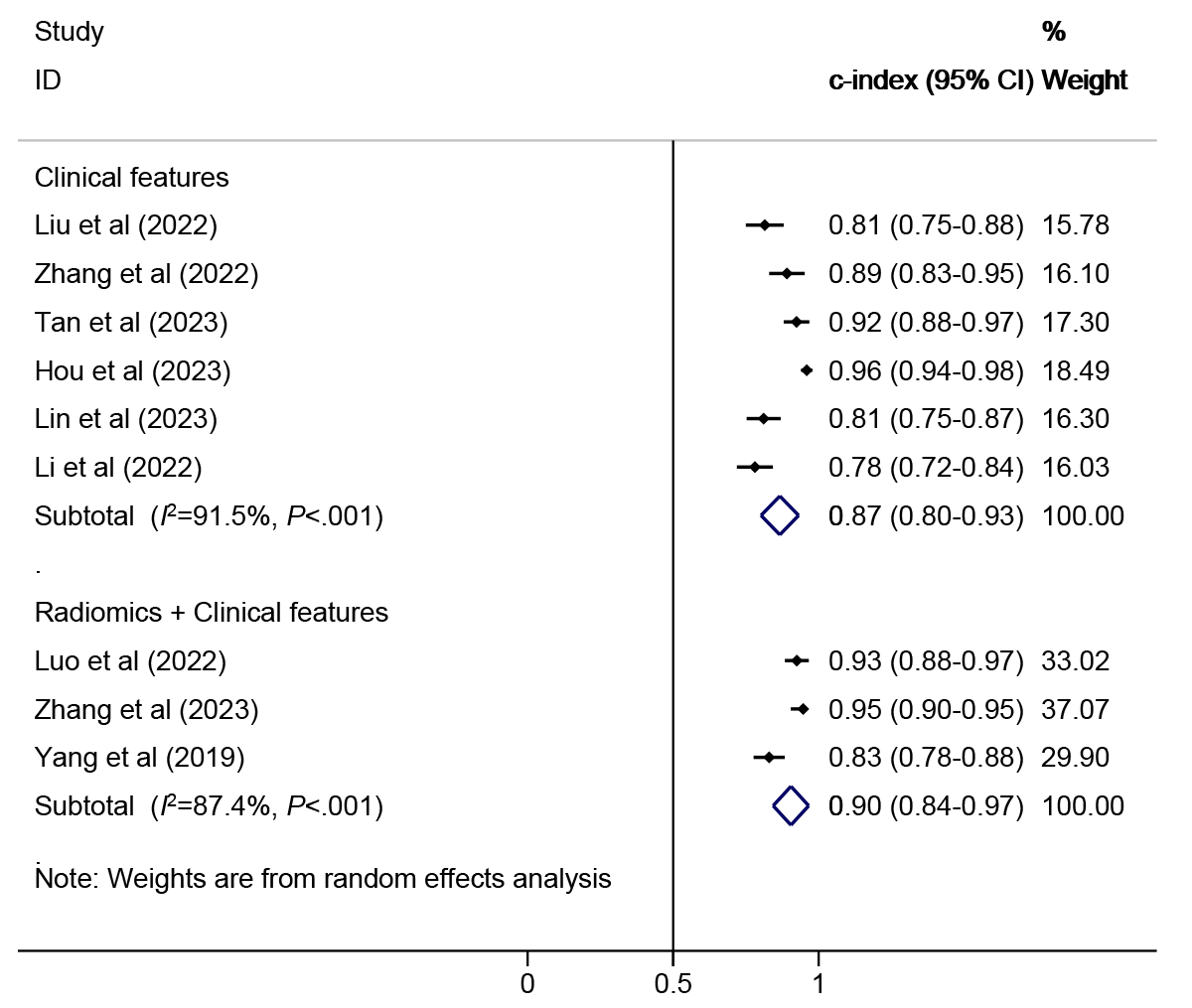

Supplement: Multimedia Appendix 5 [file jmir-v28-e78203-s005.png]

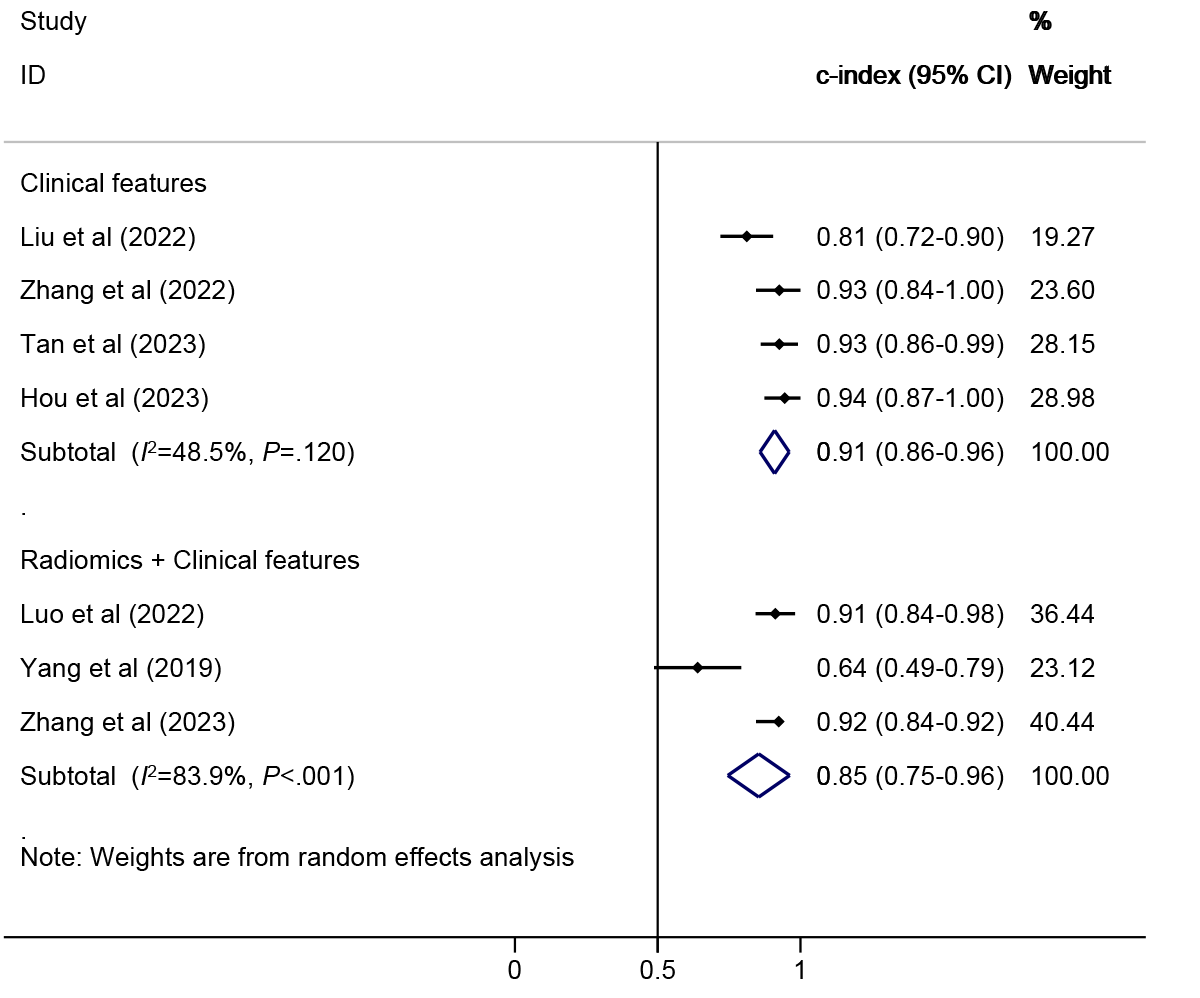

Supplement: Multimedia Appendix 6 [file jmir-v28-e78203-s006.png]

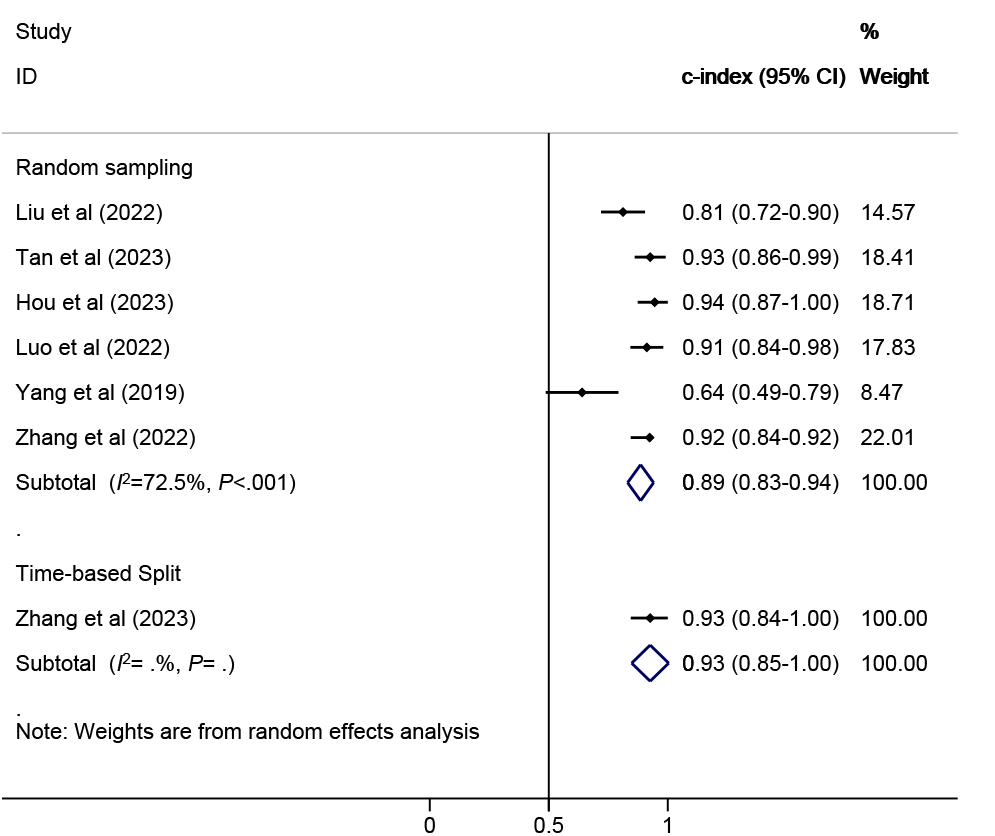

Supplement: Multimedia Appendix 7 [file jmir-v28-e78203-s007.png]
